# Xylopine Induces Oxidative Stress and Causes G_2_/M Phase Arrest, Triggering Caspase-Mediated Apoptosis by p53-Independent Pathway in HCT116 Cells

**DOI:** 10.1155/2017/7126872

**Published:** 2017-12-06

**Authors:** Luciano de Souza Santos, Valdenizia Rodrigues Silva, Leociley Rocha Alencar Menezes, Milena Botelho Pereira Soares, Emmanoel Vilaça Costa, Daniel Pereira Bezerra

**Affiliations:** ^1^Gonçalo Moniz Institute, Oswaldo Cruz Foundation (IGM-FIOCRUZ/BA), Salvador, BA, Brazil; ^2^Department of Chemistry, Federal University of Sergipe, São Cristóvão, SE, Brazil; ^3^Center of Biotechnology and Cell Therapy, Hospital São Rafael, 41253-190 Salvador, BA, Brazil; ^4^Department of Chemistry, Federal University of Amazonas, Manaus, AM, Brazil

## Abstract

Xylopine is an aporphine alkaloid that has cytotoxic activity to cancer cells. In this study, the underlying mechanism of xylopine cytotoxicity was assessed in human colon carcinoma HCT116 cells. Xylopine displayed potent cytotoxicity in different cancer cell lines in monolayer cultures and in a 3D model of cancer multicellular spheroids formed from HCT116 cells. Typical morphology of apoptosis, cell cycle arrest in the G_2_/M phase, increased internucleosomal DNA fragmentation, loss of the mitochondrial transmembrane potential, and increased phosphatidylserine externalization and caspase-3 activation were observed in xylopine-treated HCT116 cells. Moreover, pretreatment with a caspase-3 inhibitor (Z-DEVD-FMK), but not with a p53 inhibitor (cyclic pifithrin-*α*), reduced xylopine-induced apoptosis, indicating induction of caspase-mediated apoptosis by the p53-independent pathway. Treatment with xylopine also caused an increase in the production of reactive oxygen/nitrogen species (ROS/RNS), including hydrogen peroxide and nitric oxide, but not superoxide anion, and reduced glutathione levels were decreased in xylopine-treated HCT116 cells. Application of the antioxidant N-acetylcysteine reduced the ROS levels and xylopine-induced apoptosis, indicating activation of ROS-mediated apoptosis pathway. In conclusion, xylopine has potent cytotoxicity to different cancer cell lines and is able to induce oxidative stress and G_2_/M phase arrest, triggering caspase-mediated apoptosis by the p53-independent pathway in HCT116 cells.

## 1. Introduction

Colon and rectal carcinoma is a major public health problem with the third highest incidence and the fourth highest mortality worldwide [1]. The most common chemotherapy used for colon and rectal carcinoma treatment include 5-fluorouracil (5-FU, combined with folinic acid), capecitabine, irinotecan, oxaliplatin, and trifluridine (combined with tipiracil); however, resistance development and severe side effects are limitations commonly associated with the use of these drugs [2].

Aporphine alkaloids are plant-derived compounds that belong to the isoquinoline class of alkaloids and possess diverse therapeutical potential, including cancer treatment, with potent cytotoxic activity to cancer cells and the ability to prevent cell proliferation and induce apoptosis and inhibition of DNA topoisomerase and epidermal growth factor receptor [3]. Moreover, the 1,2-methylenedioxy and methylation of nitrogen have been reported as important pharmacophoric groups for the cytotoxicity of aporphine alkaloids [4, 5].

**Figure 1:**
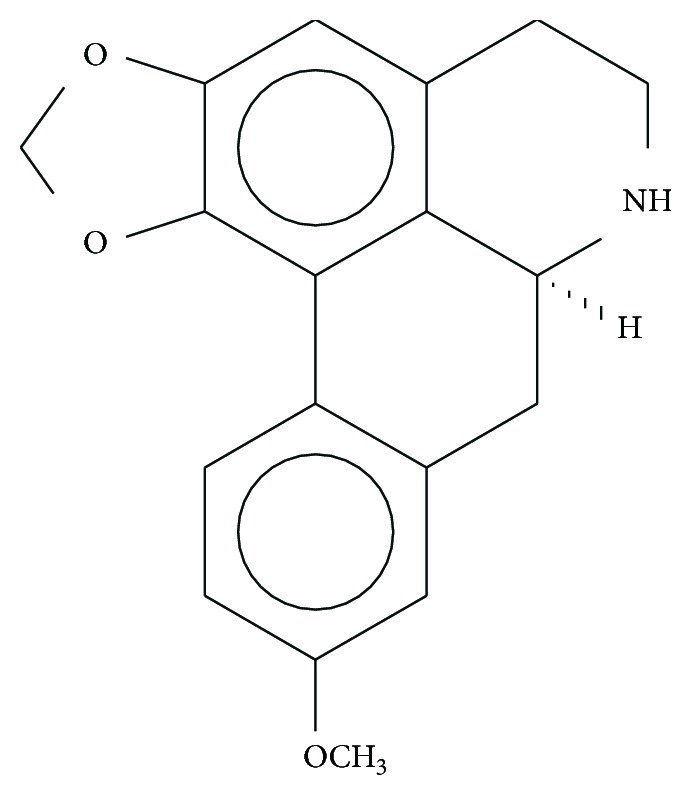
Chemical structure of xylopine.

Xylopine (Figure 1) is an aporphine alkaloid found in the stem of *Xylopia laevigata* (Annonaceae). In our previous studies, we identified xylopine as a potent cytotoxic agent that causes G_2_/M cell cycle arrest and apoptosis in human hepatocellular carcinoma HepG2 cells [6]. However, the mechanisms of action of xylopine in cancer cells have not been clearly demonstrated. In this study, the underlying mechanism of xylopine cytotoxicity was assessed in human colon carcinoma (HCT116) cells.

## 2. Material and Methods

### 2.1. Xylopine Isolation

The stem of *X. laevigata* was collected in “Serra de Itabaiana” between Itabaiana and Areia Branca cities (coordinates: 10°44′50″S, 37°20′24″W), Sergipe, Brazil, in February 2013. The identity of the plant was confirmed by Dr. Ana Paula do N. Prata, Department of Biology, Federal University of Sergipe, Brazil, and a voucher specimen (number 26805) has been deposited in the Herbarium of the Federal University of Sergipe. The dried and powdered stem of *X. laevigata* (1.4 kg) was successively extracted with hexane followed by methanol, to yield hexane (18.8 g) and methanol (87.8 g) extracts. Xylopine was isolated from the methanol extract as previously described [6].

### 2.2. Cells

MCF7 (human breast carcinoma), HCT116 (human colon carcinoma), HepG2 (human hepatocellular carcinoma), SCC-9 (human oral squamous cell carcinoma), HSC-3 (human oral squamous cell carcinoma), HL-60 (human promyelocytic leukemia), K-562 (human chronic myelogenous leukemia), B16-F10 (murine melanoma), MRC-5 (human lung fibroblast), WT SV40 MEF (wild-type immortalized mouse embryonic fibroblast), and BAD KO SV40 MEF (BAD gene knockout immortalized mouse embryonic fibroblast) cell lines were obtained from the American Type Culture Collection (ATCC, Manassas, VA, USA). Cells were cultured in complete medium with appropriate supplements as recommended by ATCC. All cell lines were tested for mycoplasma using the Mycoplasma Stain Kit (Sigma-Aldrich) to validate the use of cells free from contamination. Primary cell culture of peripheral blood mononuclear cells (PBMC) was obtained by standard Ficoll density protocol. The Research Ethics Committee of the Oswaldo Cruz Foundation (Salvador, BA, Brazil) approved the experimental protocol (number 031019/2013). Cell viability was examined using trypan blue exclusion assay for all experiments.

### 2.3. Cytotoxic Activity Assay

Cell viability was quantified using the alamarBlue assay according to Ahmed et al. [7]. Cells were inserted in 96-well plates for all experiments (7 × 10^4^ cells/mL for adherent cells or 3 × 10^5^ cells/mL for suspended cells in 100 *μ*L of medium). After 24 h, xylopine was dissolved in dimethyl sulfoxide (DMSO) and added to each well and incubated for 72 h. Doxorubicin (purity ≥ 95%, doxorubicin hydrochloride, Laboratory IMA S.A.I.C., Buenos Aires, Argentina) and oxaliplatin (Sigma-Aldrich Co., Saint Louis, MO, USA) were used as positive controls. Four (for cell lines) or 24 h (for PBMCs) before the end of incubation, 20 *μ*L of a stock solution (0.312 mg/mL) of the alamarBlue (resazurin, Sigma-Aldrich Co., Saint Louis, MO, USA) was added to each well. Absorbance at 570 nm and 600 nm was measured using the SpectraMax 190 Microplate Reader (Molecular Devices, Sunnyvale, CA, USA), and the drug effect was quantified as the percentage of control absorbance.

### 2.4. 3D Multicellular Spheroids Culture

HCT116 cells were cultivated in three-dimensional (3D) multicellular spheroids. Briefly, 100 *μ*L of a solution of cells (0.5 × 10^6^ cells/mL) was inserted in 96-well plates with a cell-repellent surface (Greiner Bio-One, Kremsmünster, Austria) and cultured in complete medium plus 3% Matrigel (BD Biosciences, San Jose, CA, USA). Spheroids with stable structures had formed after three days. Then, the spheroids were exposed to a range of drug concentrations for 72 h, after which the cell viability was quantified by alamarBlue assay as described above.

### 2.5. Morphological Analysis

To evaluate alterations in morphology, cells were cultured under coverslip and stained with May-Grünwald-Giemsa. Morphological changes were examined by light microscopy using Image-Pro software.

### 2.6. Internucleosomal DNA Fragmentation and Cell Cycle Distribution

Cells were harvested in a permeabilization solution containing 0.1% Triton X-100 (Sigma Chemical Co., St Louis, MO, USA), 2 *μ*g/mL propidium iodide (Sigma Chemical Co.), 0.1% sodium citrate, and 100 *μ*g/mL RNAse (Sigma Chemical Co.) and incubated in the dark for 15 min at room temperature [8]. Finally, cell fluorescence was measured by flow cytometry on a BD LSRFortessa cytometer using the BD FACSDiva software (BD Biosciences) and FlowJo software 10 (FlowJo, LCC, Ashland, OR, USA).

### 2.7. Annexin V/PI Staining Assay

For apoptosis detection, we used the FITC Annexin V Apoptosis Detection Kit I (BD Biosciences), and the analysis was performed according to the manufacturer's instructions. The cell fluorescence was determined by flow cytometry as described above. The protection assays using a caspase-3 inhibitor (Z-DEVD-FMK, BD Biosciences), a p53 inhibitor (cyclic pifithrin-*α*, Cayman Chemical, Ann Arbor, MI, USA), and an antioxidant N-acetyl-L-cysteine (NAC, Sigma-Aldrich Co.) were performed. In brief, the cells were pretreated for 2 h with 50 *μ*M Z-DEVD-FMK and 10 *μ*M cyclic pifithrin-*α* and for 1 h with 5 mM NAC, followed by incubation with 14 *μ*M xylopine for 48 h. The cells were then trypsinized and the FITC Annexin V Apoptosis Detection assay was conducted as described above.

### 2.8. Measurement of the Mitochondrial Transmembrane Potential

Mitochondrial transmembrane potential was determined by the retention of the dye rhodamine 123 [9]. Cells were incubated with rhodamine 123 (5 *μ*g/mL, Sigma-Aldrich Co.) at room temperature for 15 min in the dark and washed with saline. The cells were then incubated again in saline for 30 min in the dark and cell fluorescence was determined by flow cytometry as described above.

### 2.9. Caspase-3 Activation Assay

A Caspase-3 Colorimetric Assay Kit (Sigma-Aldrich Co.) was used to investigate caspase-3 activation in xylopine-treated HCT116 cells based on the cleavage of Ac-DEVD-pNA and the analysis was performed according to the manufacturer's instructions.

### 2.10. Measurement of Cellular Reactive Oxygen Species Levels

The levels of reactive oxygen species (ROS) were measured according to previously described [10] using 2′,7′-dichlorofluorescin diacetate (DCF-DA) (Sigma-Aldrich Co.). In brief, cells were treated with xylopine for 1 and 3 h. Then, the cells were collected, washed with saline, and resuspended in FACS tubes with saline containing 5 *μ*M DCF-DA for 30 min. Finally, the cells were washed with saline and the cell fluorescence was determined by flow cytometry as described above. The protection assay using the antioxidant NAC and catalase (Sigma-Aldrich Co.) was performed. In brief, the cells were pretreated for 1 h with 5 mM NAC or 2000 UI catalase and then incubated with xylopine in the established concentration for 1 h. The cells were then trypsinized and the ROS levels were measured as described above.

### 2.11. Measurement of Cellular Superoxide Anion Level

Hydroethidine (Sigma-Aldrich Co.) was used to detect cellular superoxide levels after 1 h of treatment with xylopine [11]. The cells were labeled with 10 *μ*M of hydroethidine for 30 min. Finally, the cells were washed with saline and the cell fluorescence was determined by flow cytometry as described above.

### 2.12. Measurement of Nitric Oxide Production

Nitric oxide generation was detected with 4-amino-5-methylamino-2′-,7′-difluofluorescein diacetate (DAF-FM diacetate) (Molecular Probes, Eugene, OR, USA) [12]. The cells were labeled with 3 *μ*M of DAF-FM diacetate for 60 minutes at 37°C. Following staining cells were washed with saline and incubated for an additional 15 minutes at 37°C to allow for complete deesterification of the intracellular diacatates. Then, the nitric oxide radical was measured by flow cytometry as described above.

### 2.13. Measurement of Cellular GSH Level

A quantification kit for reduced glutathione (L-*γ*-glutamyl-L-cysteinyl-glycine, GSH, Sigma-Aldrich Co.) was used to investigate cellular GSH level in xylopine-treated HCT116 cells and the analysis was performed according to the manufacturer's instructions.

### 2.14. DNA Intercalation Assay

DNA intercalation was assessed by examining the ability of xylopine to displace ethidium bromide from calf thymus DNA (ctDNA, Sigma-Aldrich Co.) [13]. The assay was conducted in 96-well plates and contained 15 *μ*g/mL ctDNA, 1.5 *μ*M ethidium bromide, and 10 or 20 *μ*M xylopine in 100 *μ*L saline solution. Doxorubicin (10 *μ*M) was used as positive control. Fluorescence was measured using excitation and emission wavelengths of 320 and 600 nm, respectively.

### 2.15. Statistical Analysis

Data are presented as mean ± S.E.M. or IC_50_ values and their 95% confidence intervals (CI 95%) obtained by nonlinear regression. Differences between experimental groups were compared using analysis of variance (ANOVA) followed by the Student–Newman–Keuls test (*p* < 0.05). All statistical analyses were performed using GraphPad (Intuitive Software for Science, San Diego, CA, USA).

## 3. Results

### 3.1. Xylopine Displays Potent Cytotoxicity in Different Cancer Cell Lines

The cytotoxicity of xylopine was assessed in eight different cancer cell lines (MCF7, HCT116, HepG2, SCC-9, HSC-3, HL-60, K-562, and B16-F10) and in two noncancer cells (MRC-5 and PBMC) using the alamarBlue assay after 72 h incubation.  Table 1 shows the results obtained. Xylopine presented IC_50_ values ranging from 6.4 to 26.6 *μ*M for cancer cells HCT116 and SCC9, respectively. Doxorubicin presented IC_50_ values ranging from 0.03 to 1.1 *μ*M for cancer cell lines B16-F10 and MCF7, respectively. Oxaliplatin presented IC_50_ values ranging from 0.1 to 5.9 *μ*M for cancer cell lines B16-F10 and MCF7, respectively. The IC_50_ value for noncancer cells was 24.1 and 18.3 *μ*M for xylopine, 1.5 and 5.2 *μ*M for doxorubicin, and 1.5 and 14.9 *μ*M for oxaliplatin for MRC5 and PBMC cells, respectively.  Table 2 shows the selectivity index obtained. For the most cancer cell lines, xylopine exhibited selectivity index similar to positive controls doxorubicin and oxaliplatin.

**Figure 2:**
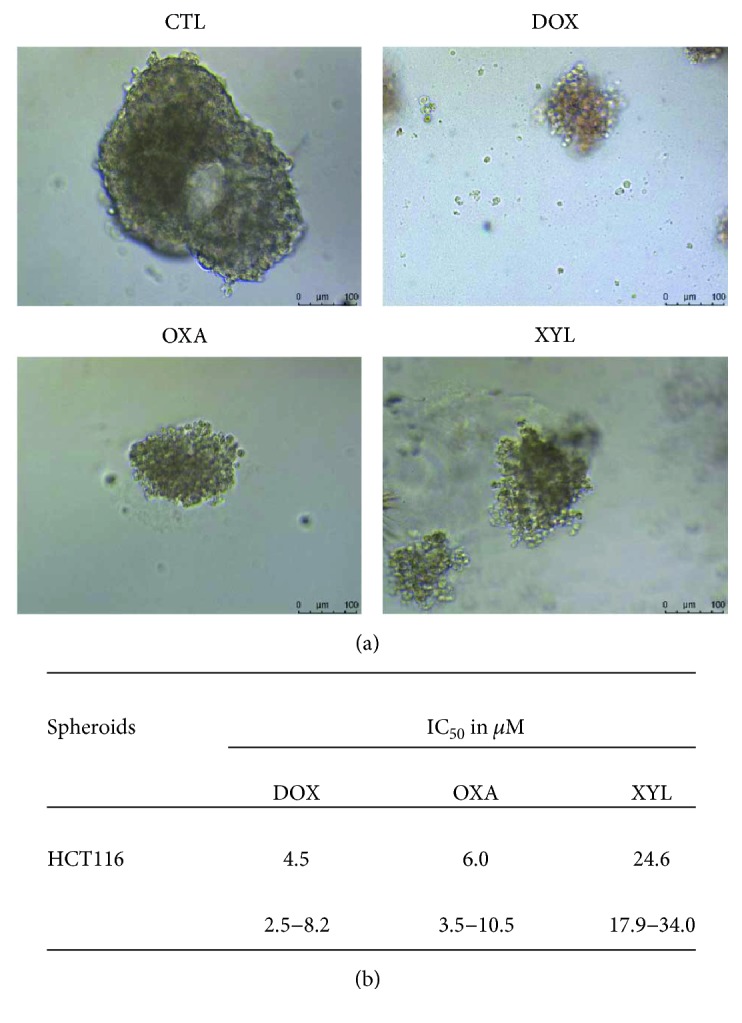
Effect of xylopine (XYL) in 3D in vitro model of cancer multicellular spheroids formed from HCT116 cells. (a) Cells examined by light microscopy (bar = 100 *μ*m). (b) IC_50_ values in *μ*M and their respective 95% confidence interval obtained by nonlinear regression from three independent experiments performed in duplicate and measured by alamarBlue assay after 72 h of incubation. The negative control (CTL) was treated with the vehicle used for diluting the compound tested. Doxorubicin (DOX) and oxaliplatin (OXA) were used as positive controls.

HCT116 was the most sensitive cell line to xylopine-induced cytotoxicity and was used as a cellular model in a new set of experiment. Furthermore, the cytotoxic effect of xylopine was then evaluated in an in vitro 3D model of cancer multicellular spheroids formed from HCT116 cells. Xylopine-treated spheroids presented morphological alterations that indicate drug permeability and cytotoxicity in the 3D culture (Figure 2(a)). The IC_50_ value of xylopine was 24.6 *μ*M after 72 h incubation (Figure 2(b)). Doxorubicin and oxaliplatin showed IC_50_ values of 4.5 and 6.0 *μ*M, respectively.

**Figure 3:**
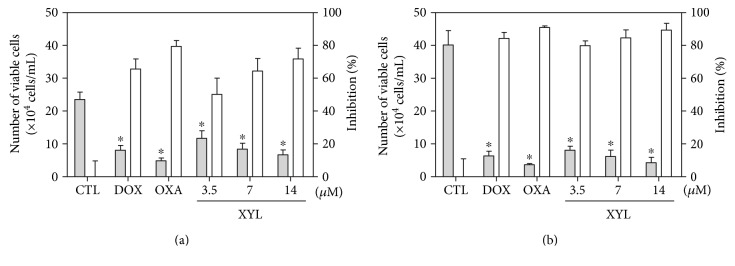
Effect of xylopine (XYL) in the cell viability of HCT116 cells determined by trypan blue staining after 24 h (a) and 48 h (b) of incubation. The gray bars represent the number of viable cells (×10^4^cells/mL), and the white bars represent cell inhibition (%). The negative control (CTL) was treated with the vehicle (0.1% DMSO) used for diluting the compound tested. Doxorubicin (DOX, 1 *μ*M) and oxaliplatin (OXA, 2.5 *μ*M) were used as positive controls. Data are presented as the mean ± S.E.M. of three independent experiments performed in duplicate. ^∗^*p* < 0.05 compared with the negative control by ANOVA followed by Student–Newman–Keuls test.

Cell viability of HCT116 cell treated with xylopine was also determined by trypan blue exclusion assay after 24 and 48 h incubation. Xylopine significantly reduced (*p* < 0.05) the number of viable cells (Figure 3). At concentrations of 3.5, 7, and 14 *μ*M, xylopine reduced the number of viable cells by 50.2, 64.4, and 71.8% after 24 h and 79.9, 84.6, and 89.4% after 48 h, respectively. No significant increase in the number of nonviable cells was observed (*p* > 0.05). Doxorubicin and oxaliplatin also reduced the number of viable cells after 24 and 48 h incubation.

### 3.2. Xylopine Induces G_2_/M Phase Arrest and Caspase-Mediated Apoptosis in HCT116 Cells

The cell cycle distribution in xylopine-treated HCT116 cells was investigated by flow cytometry after 24 and 48 h incubation.  Table 3 shows the obtained cell cycle distribution. All DNA that was subdiploid in size (sub-G_0_/G_1_) was considered fragmented. At all concentrations, xylopine treatment resulted in a significant increase in the number of cells in G_2_/M phase compared to the negative control (30.7% at control against 57.2, 58.5, and 54.0% at 3.5, 7, and 14 *μ*M xylopine after 24 h incubation and 23.8% at control against 52.0, 52.7, and 40.8% at the same concentration of xylopine after 48 h incubation, resp.). The G_2_/M phase block was followed by an increase in the internucleosomal DNA fragmentation (*p* < 0.05). Doxorubicin and oxaliplatin also caused cell cycle block at the phase G_2_/M, which was also followed by internucleosomal DNA fragmentation.

**Figure 4:**
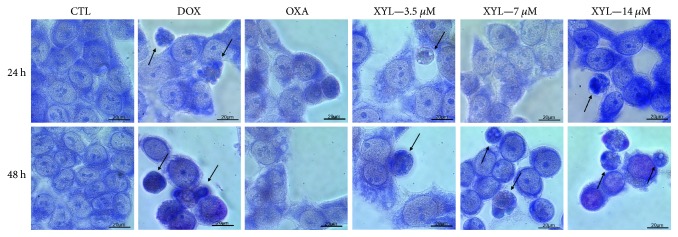
Effect of xylopine (XYL) in the morphologic analysis of HCT116 cells after 24 and 48 h incubation. The cells were stained with May-Grünwald-Giemsa and examined by light microscopy (bar = 20 *μ*m). Arrows indicated cells with a reduction in the cell volume, chromatin condensation, or fragmented DNA. The negative control (CTL) was treated with the vehicle (0.1% DMSO) used for diluting the compound tested. Doxorubicin (DOX, 1 *μ*M) and oxaliplatin (OXA, 2.5 *μ*M) were used as positive controls.

**Figure 5:**
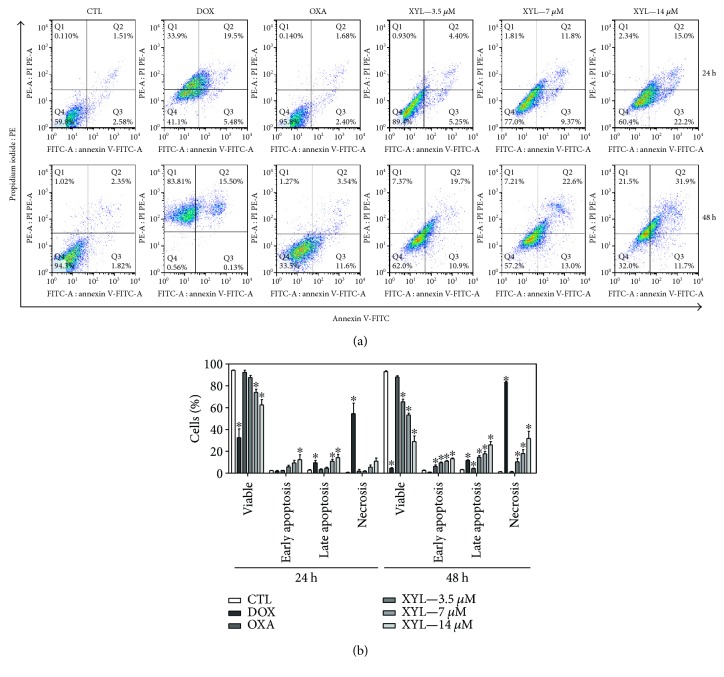
Effect of xylopine (XYL) in the induction of apoptosis in HCT116 cells determined by flow cytometry using annexin V-FITC/PI staining after 24 and 48 h incubation. (a) Representative flow cytometric dot plots showing the percentage of cells in viable, early apoptotic, late apoptotic, and necrotic stages. (b) Quantification of the cell viability. The negative control (CTL) was treated with the vehicle (0.1% DMSO) used for diluting the compound tested. Doxorubicin (DOX, 1 *μ*M) and oxaliplatin (OXA, 2.5 *μ*M) were used as positive controls. Data are presented as the mean ± S.E.M. of three independent experiments performed in duplicate. Ten thousand events were evaluated per experiment and cellular debris was omitted from the analysis. ^∗^*p* < 0.05 compared with the negative control by ANOVA followed by Student–Newman–Keuls test.

**Figure 6:**
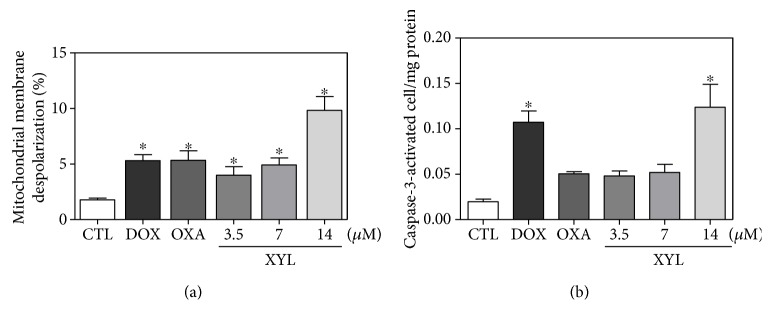
Effect of xylopine (XYL) in the caspase-3 activity and mitochondrial membrane potential in HCT116 cells. (a) Mitochondrial membrane potential determined by flow cytometry using rhodamine 123 staining after 24 h incubation. (b) Caspase-3 activity determined by colorimetric assay after 48 h incubation. The negative control (CTL) was treated with the vehicle (0.1% DMSO) used for diluting the compound tested. Doxorubicin (DOX, 1 *μ*M) and oxaliplatin (OXA, 2.5 *μ*M) were used as positive controls. Data are presented as the mean ± S.E.M. of three independent experiments performed in duplicate. For flow cytometry analysis, 10,000 events were evaluated per experiment and cellular debris was omitted from the analysis. ^∗^*p* < 0.05 compared with the negative control by ANOVA followed by Student–Newman–Keuls test.

**Figure 7:**
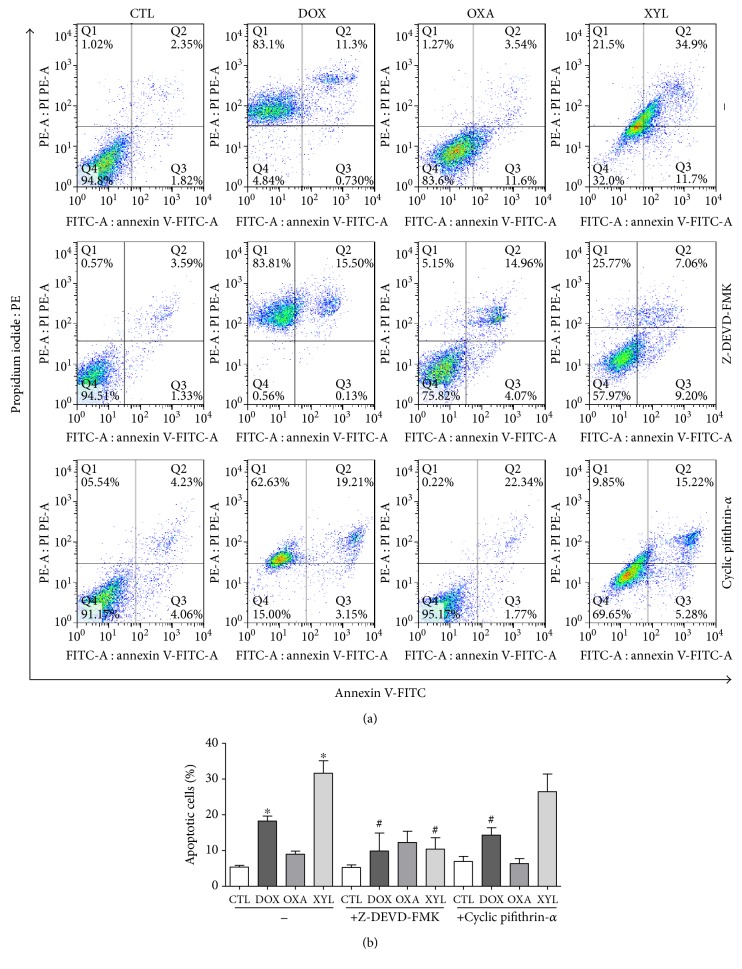
Effect of the caspase-3 inhibitor (Z-DEVD-FMK) and p53 inhibitor (cyclic pifithrin-*α*) in xylopine-induced apoptosis in HCT116 cells determined by flow cytometry using annexin V-FITC/PI staining. (a) Representative flow cytometric dot plots showing the percentage of cells in viable, early apoptotic, late apoptotic, and necrotic stages. (b) Quantification of apoptotic cells. The cells were pretreated for 2 h with 50 *μ*M Z-DEVD-FMK and 10 *μ*M cyclic pifithrin-*α* and then incubated with 14 *μ*M xylopine (XYL) for 48 h. The negative control (CTL) was treated with the vehicle (0.1% DMSO) used for diluting the compound tested. Doxorubicin (DOX, 1 *μ*M) and oxaliplatin (OXA, 2.5 *μ*M) were used as positive controls. Data are presented as the mean ± S.E.M. of three independent experiments performed in duplicate. Ten thousand events were evaluated per experiment, and cellular debris was omitted from the analysis. ^∗^*p* < 0.05 compared with the negative control by ANOVA followed by Student–Newman–Keuls test. ^#^*p* < 0.05 compared with the respective treatment without inhibitor by ANOVA followed by Student–Newman–Keuls test.

Cell morphology of xylopine-treated HCT116 cells presented a reduction in the cell volume, chromatin condensation, and fragmentation of the nuclei (Figure 4). Doxorubicin and oxaliplatin also induced cell shrinkage, chromatin condensation, and nuclear fragmentation. Apoptosis induction was assessed using the annexin V/PI double stain by flow cytometry in xylopine-treated HCT116 cells (Figure 5). Xylopine significantly increased the early and late apoptosis (*p* < 0.05). A significant increase in necrotic cells was observed in xylopine-treated HCT116 cells after 48 h incubation (*p* < 0.05). Xylopine also induced mitochondrial depolarization (*p* < 0.05) in HCT116 cells after 24 h incubation (Figure 6(a)) and increased caspase-3 activation (*p* < 0.05) after 48 h incubation (Figure 6(b)). Moreover, cotreatment with a caspase-3 inhibitor (Z-DEVD-FMK), but not with a p53 inhibitor (cyclic pifithrin-*α*), prevented the xylopine-induced increasing apoptosis (Figures 7(a) and 7(b)). On the other hand, the IC_50_ values for xylopine were 6.4 *μ*M for the BAD gene knockout immortalized mouse embryonic fibroblast (BAD KO SV40 MEF) cell line, while 8.0 *μ*M was for wild-type immortalized mouse embryonic fibroblast (WT SV40 MEF) cell line, suggesting that BAD gene is not essential for xylopine-induced cytotoxicity. Doxorubicin presents IC_50_ values of 0.4 and 0.03 *μ*M, while 5-FU presents IC_50_ values of 7.3 and 1.7 *μ*M on BAD KO SV40 MEF and WT SV40 MEF cell lines, respectively.

### 3.3. Xylopine Causes ROS-Mediated Apoptosis in HCT116 Cells

**Figure 8:**
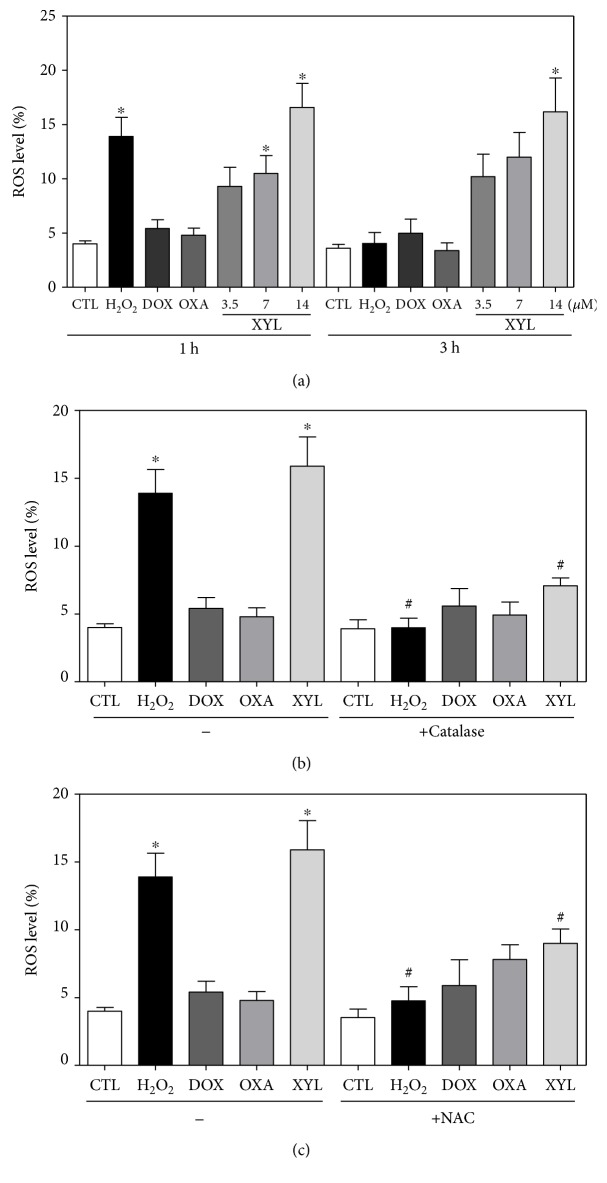
Effect of xylopine (XYL) in the levels of reactive oxygen species (ROS) of HCT116 cells and protection by N-acetyl-L-cysteine (NAC) and catalase determined by flow cytometry using DCF-DA staining. (a) ROS levels of HCT116 cells after 1 and 3 h incubation. (b) ROS levels of HCT116 cells pretreated with the antioxidant NAC and then treated with xylopine. (c) ROS levels of HCT116 cells pretreated with the antioxidant catalase and then treated with xylopine. For the protection assay, the cells were pretreated for 1 h with 5 mM NAC or 2000 UI catalase and then incubated with 14 *μ*M xylopine for 1 h. The negative control (CTL) was treated with the vehicle (0.1% DMSO) used for diluting the compound tested. Hydrogen peroxide (H_2_O_2_, 200 *μ*M), doxorubicin (DOX, 1 *μ*M), and oxaliplatin (OXA, 2.5 *μ*M) were used as positive controls. Data are presented as the mean ± S.E.M. of three independent experiments performed in duplicate or triplicate. Ten thousand events were evaluated per experiment, and cellular debris was omitted from the analysis. ^∗^*p* < 0.05 compared with the negative control by ANOVA followed by Student–Newman–Keuls test. ^#^*p* < 0.05 compared with the respective treatment without inhibitor by ANOVA followed by Student–Newman–Keuls test.

**Figure 9:**
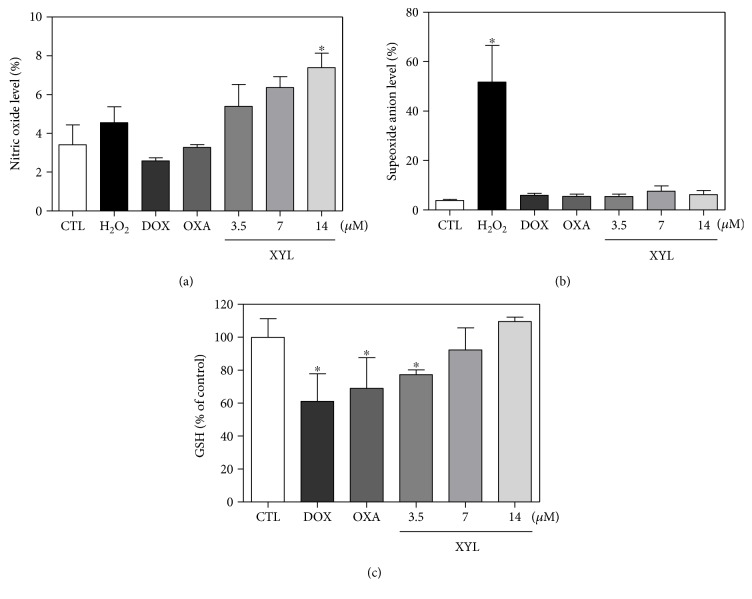
Effect of xylopine (XYL) in the levels of reactive oxygen species (ROS) and reduced glutathione (GSH) of HCT116 cells after 1 h incubation. (a) Nitric oxide level of HCT116 cells determined by flow cytometry using DAF-FM diacetate staining. (b) Superoxide anion level of HCT116 cells determined by flow cytometry using hydroethidine staining. (c) GSH level of HCT116 cells determined by colorimetric assay. The negative control (CTL) was treated with the vehicle (0.1% DMSO) used for diluting the compound tested. Hydrogen peroxide (H_2_O_2_, 200 *μ*M), doxorubicin (DOX, 1 *μ*M), and oxaliplatin (OXA, 2.5 *μ*M) were used as positive controls. Data are presented as the mean ± S.E.M. of three independent experiments performed in duplicate. For flow cytometry analysis, 10,000 events were evaluated per experiment and cellular debris was omitted from the analysis. ^∗^*p* < 0.05 compared with the negative control by ANOVA followed by Student–Newman–Keuls test.

**Figure 10:**
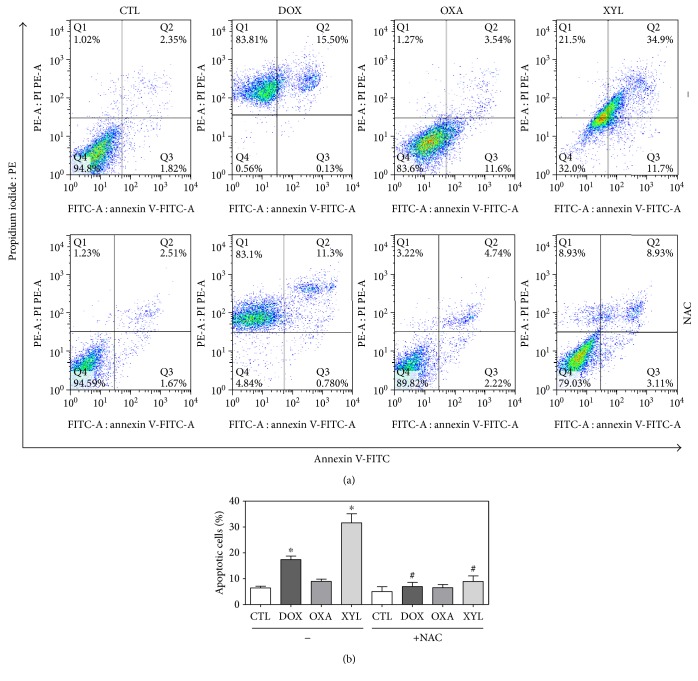
Effect of the antioxidant N-acetyl-L-cysteine (NAC) in xylopine-induced apoptosis in HCT116 cells determined by flow cytometry using annexin V-FITC/PI staining. (a) Representative flow cytometric dot plots showing the percentage of cells in viable, early apoptotic, late apoptotic, and necrotic stages. (b) Quantification of apoptotic cells. The cells were pretreated for 1 h with 5 mM NAC and then incubated with 14 *μ*M xylopine (XYL) for 48 h. The negative control (CTL) was treated with the vehicle (0.1% DMSO) used for diluting the compound tested. Doxorubicin (DOX, 1 *μ*M) and oxaliplatin (OXA, 2.5 *μ*M) were used as positive controls. Data are presented as the mean ± S.E.M. of three independent experiments performed in duplicate. Ten thousand events were evaluated per experiment, and cellular debris was omitted from the analysis. ^∗^*p* < 0.05 compared with the negative control by ANOVA followed by Student–Newman–Keuls test. ^#^*p* < 0.05 compared with the respective treatment without inhibitor by ANOVA followed by Student–Newman–Keuls test.

The effect of xylopine in intracellular reactive oxygen/nitrogen species (ROS/RNS) levels was investigated in HCT116 cells through flow cytometry. Treatment with xylopine for 1 and 3 h caused an increase in the ROS levels (Figure 8(a)), and the cotreatment with the antioxidant NAC prevented the xylopine-induced increase in the intracellular ROS level (Figure 8(b)). Cotreatment with catalase, which induces decomposition of hydrogen peroxide, also prevented xylopine-induced increase in intracellular ROS levels that indicate the production of hydrogen peroxide induced by xylopine (Figure 8(c)). Using fluorescent probe specific for individual ROS/RNS, we found that xylopine increases nitric oxide (Figure 9(b)), but not superoxide anion (Figure 9(a)), in HCT116 cells. Furthermore, GSH levels were decreased in xylopine-treated cells (Figure 9(c)). The cotreatment with NAC also prevented the xylopine-induced increase of the cell death by apoptosis (Figures 10(a) and 10(b)).

### 3.4. Xylopine Does Not Induce DNA Intercalation

DNA intercalation was evaluated by examining the ability of xylopine to displace ethidium bromide from ctDNA and thus decreasing the fluorescence intensity of ethidium bromide. Xylopine was not able to decrease the ethidium bromide fluorescence, indicating that it is not a strong DNA intercalator. Doxorubicin, a potent DNA intercalator, significantly reduced the fluorescence intensity (data not shown).

## 4. Discussion

In the present study, we report for the first time that xylopine induces oxidative stress and causes G_2_/M phase arrest triggering caspase-mediated apoptosis in HCT116 cells. Previous works with xylopine reported that it induces potent cytotoxicity to cancer cells, but its mechanism of action has been not described [6].

Diverse aporphine alkaloids have been reported to exhibit cytotoxic effect on various types of human cancer cells, including acutiaporberine, anonaine, artabotrine, lysicamine, magnoflorine, norglaucine, norpurpureine, calycinine, and liriodenine [6, 14–20]. Acutiaporberine induces apoptosis in human non-small-cell lung cancer PLA-801 cells and human lung cancer 95-D cells, accompanied by downregulation of the bcl-2 gene and upregulation of the bax gene [14, 15]. Liriodenine inhibits human lung adenocarcinoma A549 cell proliferation and blocks the cell cycle progression at the G_2_/M phase, accompanied by a reduction of G_1_ cyclin D1 and accumulation of G_2_ cyclin B1, and the enzymatic activity of the cyclin B1/cyclin-dependent kinase 1 complex was reduced in liriodenine-treated cells. Activation of caspases and induction of apoptosis were also observed in liriodenine-treated A549 cells [16]. Herein, xylopine blocked cell cycle progression at the G_2_/M phase and triggered caspase-mediated apoptosis pathway in HCT116 cells, as observed by internucleosomal DNA fragmentation, externalization of phosphatidylserine, loss of mitochondrial transmembrane potential, and activation of caspase-3. Additionally, xylopine-induced apoptosis was prevented by pretreatment with a caspase-3 inhibitor, but not with a p53 inhibitor.

ROS/RNS are toxic products of cellular metabolism and are involved in cellular apoptosis by both the extrinsic cell death receptor pathway and the intrinsic mitochondrial cell death pathway. ROS include free radicals deprived of oxygen_,_ including superoxide anions (O_2_^•−^), hydroxyl radicals (HO^•^), peroxyl (RO_2_^•^), alkoxyl (RO^•^), and nonradical species deprived of oxygen, including hydrogen peroxide (H_2_O_2_), whereas RNS include mainly nitric oxide (^•^NO). Moreover, glutathione is usually presented as a reduced form (GSH), but GSH can be converted into an oxidized form (GSSG) by stimulation of oxidative stress, and decreased cellular GSH levels are associated with ROS-mediated apoptosis [21–25]. Xylopine induces oxidative stress, including the increase of the levels of nitric oxide and hydrogen peroxide, but not superoxide anion, in HCT116 cells. Depletion of cellular GSH was also observed in xylopine-treated cells. Moreover, pretreatment with the antioxidant NAC prevented xylopine-induced apoptosis, indicating ROS-mediated apoptosis pathway. The aporphine alkaloids anonaine, glaucine, and norglaucine were previously reported as inductors of oxidative stress [18]. In addition, liriodenine/valproic acid combination treatment enhances ROS production and intracellular GSH depletion [26].

Some aporphine alkaloids are DNA intercalator agents, including liriodenine and dicentrine, which can induce inhibition of DNA topoisomerases as a mechanism of cytotoxicity [27]. Herein, DNA intercalation ability of xylopine was assessed in ctDNA; however, xylopine failed to induce DNA intercalation.

## 5. Conclusion

In conclusion, xylopine has potent cytotoxicity to different cancer cell lines and induces oxidative stress and causes G_2_/M phase arrest triggering caspase-mediated apoptosis by the p53-independent pathway in HCT116 cells.

## Figures and Tables

**Table 1:** Cytotoxic activity of xylopine (XYL).

Cells	Histological type	IC_50_ in *μ*M		
		DOX	OXA	XYL
Cancer cells				
MCF7	Human breast carcinoma	1.1	5.9	12.0
		0.3–3.5	3.5–9.9	6.1–23.6
HCT116	Human colon carcinoma	0.1	4.1	6.4
		0.04–0.11	2.7–6.4	5.1–8.2
HepG2	Human hepatocellular carcinoma	0.1	1.0	9.4
		0.04–0.11	0.2-3.9	6.0–14.8
SCC9	Human oral squamous cell carcinoma	0.5	N.d.	26.6
		0.4–0.7		21.9–32.1
HSC3	Human oral squamous cell carcinoma	0.3	3.3	15.7
		0.2–0.4	1.4–7.8	10.2–24.3
HL-60	Human promyelocytic leukemia	0.3	0.4	18.5
		0.3–0.4	0.1–3.8	16.0–21.3
K562	Human chronic myelogenous leukemia	0.3	0.9	7.8
		0.2–0.5	0.1–9.7	6.1–9.9
B16-F10	Murine melanoma	0.03	0.1	9.6
		0.02–0.07	0.03–1.3	8.0–11.4
Noncancer cells				
MRC5	Human lung fibroblast	1.5	1.5	24.1
		1.2–2.0	0.9–2.8	20.8–28.3
PBMC	Human peripheral blood mononuclear cells	5.2	14.9	18.3
		2.4–11.4	8.9–24.8	11.0–29.0

Data are presented as IC_50_ values in *μ*M and their respective 95% confidence interval obtained by nonlinear regression from at the least three independent experiments performed in duplicate and measured by alamarBlue assay after 72 h incubation. Doxorubicin (DOX) and oxaliplatin (OXA) were used as positive controls. N.d.: not determined.

**Table 2:** Selectivity index of xylopine (XYL).

Cancer cells	Noncancer cells					
	MRC5			PBMC		
	DOX	OXA	XYL	DOX	OXA	XYL
MCF7	1.4	0.3	2	4.7	2.5	1.5
HCT116	15	0.4	3.8	52	3.6	2.9
HepG2	15	1.5	2.6	52	14.9	2
SCC-9	3	N.d.	0.9	10.4	N.d.	0.7
HSC-3	5	0.5	1.5	17.3	4.5	1.2
HL-60	5	3.8	1.3	17.3	37.3	1
K-562	5	1.7	3.1	17.3	16.6	2.4
B16-F10	50	15	2.5	173.3	149	1.9

Data are presented the selectivity index (SI) calculated using the following formula: SI = IC_50_(noncancer cells)/IC_50_(cancer cells). Cancer cells: MCF7 (human breast carcinoma), HCT116 (human colon carcinoma), HepG2 (human hepatocellular carcinoma), SCC-9 (human oral squamous cell carcinoma), HSC-3 (human oral squamous cell carcinoma), HL-60 (human promyelocytic leukemia), K-562 (human chronic myelogenous leukemia), and B16-F10 murine melanoma. Noncancer cells: MRC-5 (human lung fibroblast) and PBMC human peripheral blood mononuclear cells. Doxorubicin (DOX) and oxaliplatin (OXA) were used as positive controls. N.d.: not determined.

**Table 3:** Effect of xylopine (XYL) in the cell cycle distribution of HCT116 cells.

Treatment	Concentration (*μ*M)	DNA content (%)			
		Sub-G_0_/G_1_	G_0_/G_1_	*S*	G_2_/M
24 h incubation					
CTL	—	3.9 ± 1.0	42.0 ± 2.5	12.6 ± 2.8	30.7 ± 4.1
DOX	1	9.7 ± 2.5	28.0 ± 6.3	10.0 ± 2.5	44.1 ± 3.4^∗^
OXA	2.5	8.8 ± 3.5	32.4 ± 3.7	13.5 ± 3.1	39.9 ± 1.5
XYL	3.5	6.2 ± 1.9	17.0 ± 7.0^∗^	24.7 ± 4.8	57.2 ± 3.5^∗^
	7	5.1 ± 1.6	14.9 ± 4.2^∗^	23.3 ± 3.7	58.5 ± 2.9^∗^
	14	11.3 ± 1.4	30.5 ± 6.0	11.1 ± 1.6	54.0 ± 6.1^∗^
48 h incubation					
CTL	—	3.3 ± 0.7	44.8 ± 1.1	13.5 ± 2.4	23.8 ± 2.8
DOX	1	18.3 ± 2.5	22.5 ± 4.0^∗^	14.9 ± 2.5	44.2 ± 4.4^∗^
OXA	2.5	17.7 ± 1.9	36.2 ± 2.6	7.6 ± 0.4	32.2 ± 4.4
XYL	3.5	15.2 ± 1.9	17.0 ± 6.3^∗^	11.1 ± 2.2	52.0 ± 6.8^∗^
	7	25.9 ± 2.5^∗^	10.3 ± 1.3^∗^	6.8 ± 2.0	52.7 ± 3.6^∗^
	14	33.4 ± 6.2^∗^	14.6 ± 2.6^∗^	9.3 ± 1.7	40.8 ± 5.7^∗^

Data are presented as the mean ± S.E.M. of three independent experiments performed in duplicate. The negative control (CTL) was treated with the vehicle (0.1% DMSO) used for diluting the compound tested. Doxorubicin (DOX) and oxaliplatin (OXA) were used as positive controls. Ten thousand events were evaluated per experiment, and cellular debris was omitted from the analysis. ^∗^*p* < 0.05 compared with the negative control by ANOVA followed by Student–Newman–Keuls test.

## Abbreviations

3D: Three-dimensional
5-FU: 5-fluorouracil
ATCC: American Type Culture Collection
ctDNA: Calf thymus DNA
DAF-FM diacetate: 4-amino-5-methylamino-2′-,7′-difluofluorescein diacetate
DCF-DA: 2′,7′-dichlorofluorescin diacetate
DMSO: Dimethyl sulfoxide
GSH: L-*γ*-glutamyl-L-cysteinyl-glycine
NAC: N-acetyl-L-cysteine
PBMC: Peripheral blood mononuclear cells
RNS: Reactive nitrogen species
ROS: Reactive oxygen species.
